# Inflammatory Knee Reaction Mimicking Septic Arthritis Following Closely Spaced Pneumococcal Vaccines in a Pediatric Patient Undergoing Immunodeficiency Evaluation

**DOI:** 10.7759/cureus.85537

**Published:** 2025-06-07

**Authors:** Silvia M Soule, Annabelle Huntsman, Swati Chandra, Tyler Thorne, Stephanie Holmes, Kristina McKinley

**Affiliations:** 1 Medical School, University of Utah School of Medicine, Salt Lake City, USA; 2 Pediatrics, University of Utah School of Medicine, Salt Lake City, USA; 3 Orthopaedics, University of Utah School of Medicine, Salt Lake City, USA; 4 Pediatric Hospital Medicine, University of Utah School of Medicine, Salt Lake City, USA

**Keywords:** immunodeficiency, inflammatory reaction, pediatric case report, pneumococcal vaccine, septic arthritis mimic, transient synovitis, vaccine-induced myositis

## Abstract

Vaccines in the pediatric population are crucial in preventing infectious diseases, but rare inflammatory responses can arise, particularly in patients with suspected immunodeficiency. This case highlights a unique inflammatory reaction mimicking septic arthritis following closely spaced pneumococcal vaccines (PPSV23) in a child undergoing immunodeficiency work-up. A previously healthy five-year-old Caucasian female with a history of recurrent bacterial infections presented with a two-day history of left knee swelling and pain after receiving two pneumococcal vaccines within a five-week interval due to a scheduling error. She received the vaccine as part of a “pneumococcal challenge” to assess for an appropriate immunological response. On initial presentation, she was febrile and unable to bear weight; with laboratory findings significant for a white blood cell (WBC) count of 13.6 x10^9^/L, erythrocyte sedimentation rate (ESR) of 26 mm/hour, and C-reactive protein (CRP) of 7.4 mg/dL. Knee joint aspiration revealed a WBC count of 13,000 x 10^9^/L with neutrophil predominance. MRI of the knee demonstrated myositis, cellulitis, and non-necrotizing fasciitis but no evidence of septic arthritis or osteomyelitis. She received empiric intravenous antibiotics, followed by oral therapy with a plan to continue antibiotics for seven days based on her clinical response. By the time of discharge, her symptoms had fully resolved. This unique case underscores the importance of careful vaccine scheduling in the setting of immunodeficiency work-up, highlighting the potential for vaccine-induced inflammatory reactions in this patient population. Clinicians should consider post-vaccination immune responses in the differential diagnosis of acute joint swelling, especially when infectious causes are not clearly identified.

## Introduction

Childhood pneumococcal vaccines have significantly reduced the global burden of invasive pneumococcal disease since their introduction [1]; however, rare adverse events following immunization can pose diagnostic challenges, particularly in vulnerable populations. While vaccine-induced joint inflammation is uncommon, it can mimic bacterial arthritis, prompting extensive infectious work-up [2]. Prior research has indicated that exaggerated immune responses to pneumococcal vaccines in the pediatric population are more frequently reported in patients with underlying conditions such as sickle-cell disease [2]. While their findings emphasize the potential for increased inflammatory responses in patients with similar conditions, this case report builds on these findings by examining a distinct clinical scenario in a pediatric patient with an undifferentiated immunodeficiency.

In the United States, the CDC recommends routine administration of pneumococcal conjugate vaccines (PCV13 or PCV15) in infancy, with the 23-valent pneumococcal polysaccharide vaccine (PPSV23) reserved for children with chronic conditions or immunocompromising states [3]. In such patients, immunologic work-ups typically include serum immunoglobulin levels, IgG subclasses, and evaluation of antibody responses to polysaccharide vaccines. 

In pediatric populations, septic arthritis often results from hematogenous spread of infection with *Staphylococcus aureus *or *Streptococcus* species, which are the most frequently isolated pathogens in patient laboratory samples [4]. This condition often presents with fever, joint swelling, pain, and inability to bear weight, requiring joint aspiration for diagnosis. Similarly, vaccine-induced adverse reactions can cause localized joint swelling, tenderness, and systemic inflammation. Both conditions can result in systemic inflammatory marker elevation, leading to diagnostic overlap [5]. Pediatric patients with undifferentiated immune dysregulation may have a heightened sensitivity to vaccine antigens, increasing their risk of inflammatory responses. This case demonstrates the importance of differentiating between post-vaccination inflammatory reactions and true infectious arthritis. Here, we present a case of a pediatric female with an inflammatory reaction of the knee following two pneumococcal vaccinations (PPSV23) within a five-week interval.

## Case presentation

A five-year-old Caucasian female child presented to the pediatric emergency department with a two-day history of left knee swelling, accompanied by pain and fever. She had a history of severe, recurrent sinus infections over the past year and was undergoing an immunodeficiency work-up at the time of presentation, raising concern for underlying conditions such as IgA or IgG subclass deficiency. This history heightened initial concern for septic arthritis and supported the decision to initiate empiric antibiotics.

On examination, the patient was mildly febrile and unable to bear weight on her left leg. Physical assessment revealed a swollen, erythematous knee with limited range of motion. Initial laboratory findings included a white blood cell (WBC) count of 13.6 x10^9^/L, erythrocyte sedimentation rate (ESR) of 26 mm/hour, and C-reactive protein (CRP) of 7.4mg/dL (Table 1), which showed an upward trend from previously obtained values.

**Table 1:** Selected laboratory and synovial fluid findings in the five-year-old female patient presenting with left knee swelling following recent PPSV23 vaccination. WBC: white blood cell count; CRP: C-reactive protein; ESR: erythrocyte sedimentation rate; RBC: red blood cell count Note: Reference ranges based on Intermountain Primary Children’s Hospital laboratory standards.

Laboratory Test	Patient Values	Reference Range	
WBC (blood)	13.6 × 10⁹/L	5.0–14.5 × 10⁹/L	
ESR	26 mm/hour	0–20 mm/hour	
CRP	7.4 mg/dL	< 0.5 mg/dL	
Synovial WBC	13,000 × 10^9^/L	< 200 cells/µL (non-inflammatory); >50,000 suggests septic	
Synovial Neutrophils	83%	< 25% (normal); >75% in septic arthritis	
Synovial RBC	303,000/mcL	< 2,000/mcL	
Gram Stain (Synovial Fluid)	1+ WBCs; no organisms	No organisms expected	
Synovial Fluid Culture	No growth after 5 days	Negative	
Blood Culture	Negative	Negative	

The complete blood count (CBC) differential showed neutrophilic predominance without a left shift or bandemia. The patient was non-weightbearing and had an elevated white blood cell count, findings that raised concern for a possible septic joint. Joint aspiration in the emergency department yielded grossly bloody and cloudy synovial fluid, with a WBC count of 13,000 x 10^9^/L with neutrophilic predominance (83% neutrophils) and an RBC count of 303,000/mcL, suggestive of either a traumatic tap or hemoarthrosis. Gram stain showed 1+ WBCs without any organisms seen, synovial fluid culture demonstrated no growth after five days (Table 1). Blood cultures were also obtained and remained negative.

**Figure 1:**
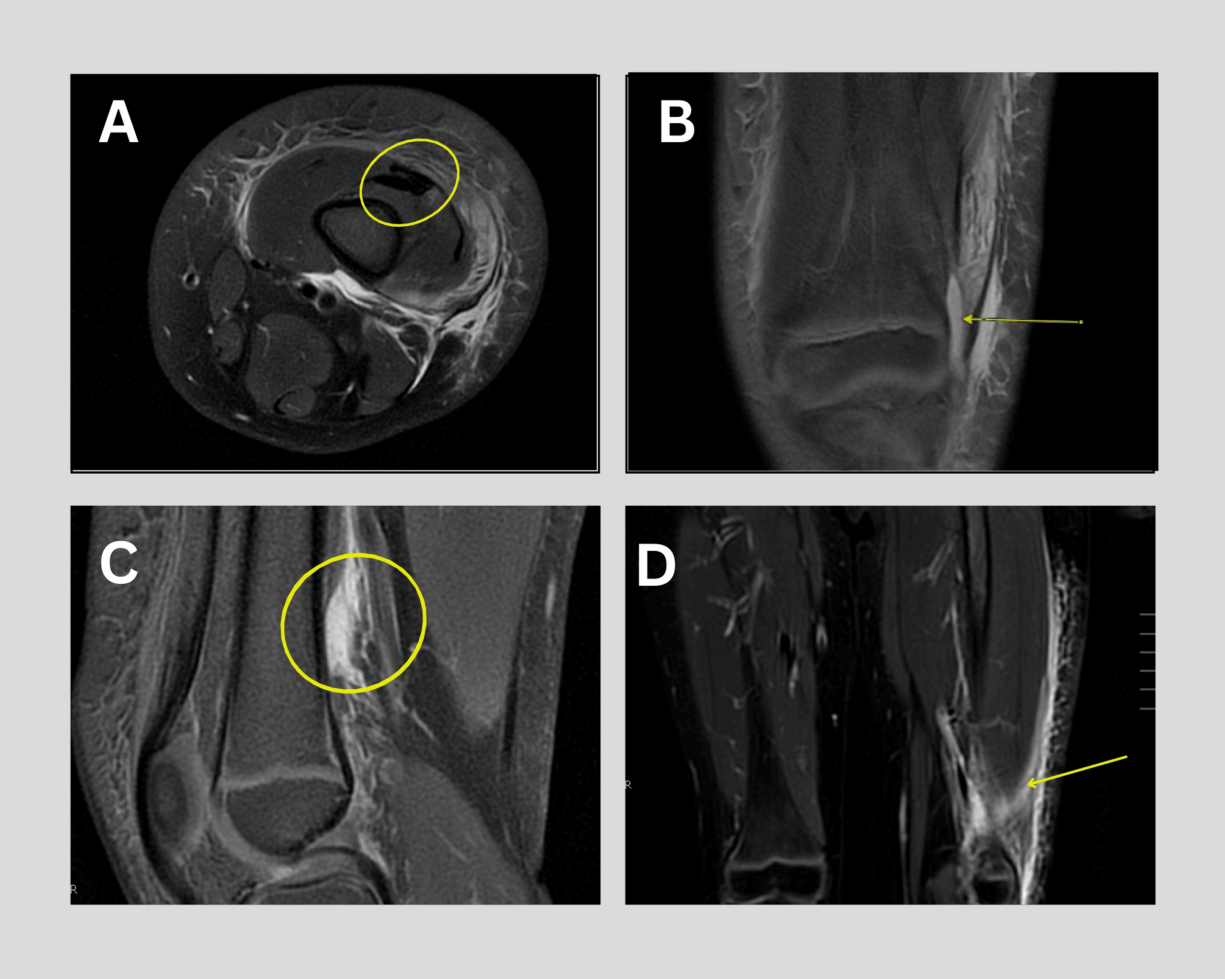
MRI of the left thigh and knee demonstrating post-vaccination inflammatory changes. (A) Axial image at the distal femur showing edema and myositis within the anterior compartment musculature; (B) Coronal image showing intramuscular fluid within the vastus lateralis with perimysial stranding; (C) Sagittal image with anterior compartment and fascial hyperintensity consistent with cellulitis and fasciitis; (D) Coronal image revealing soft tissue inflammation extending into the lateral posterior compartment.

MRI of the left lower extremity demonstrated myositis, subcutaneous edema, and fascial hyperintensity, but no evidence of joint effusion, synovial enhancement, or osteomyelitis (Figure 1). These findings were consistent with inflammation localized to the subcutaneous and muscular planes, rather than intra-articular pathology.

Given the absence of joint effusion on imaging and negative preliminary culture results, both the orthopaedic and general surgery teams advised against surgical intervention. The patient was admitted for further monitoring and was started on intravenous cefazolin (Ancef) for empiric antibiotic coverage due to initial concern for septic arthritis.

Additional history obtained from the patient's parents during hospitalization revealed that she had inadvertently received two doses of pneumococcal vaccine (PPSV23) within a five-week interval as part of immunodeficiency work-up. Two days prior to symptom onset, she received a second dose of the PPSV23 in her left thigh, which was inadvertently administered within five weeks of previously receiving the same vaccine. This detail, initially unknown to the medical team, shifted the working diagnosis toward a vaccine-induced inflammatory reaction. Following clinical improvement and negative infectious work-up, she was transitioned to oral cephalexin (250 mg/5 mL oral liquid, three times daily) to complete a seven-day empiric course. She was also treated symptomatically with non-steroidal anti-inflammatory drugs (NSAIDs) (Ibuprofen 240 mg every six hours as needed for pain).

**Figure 2:**
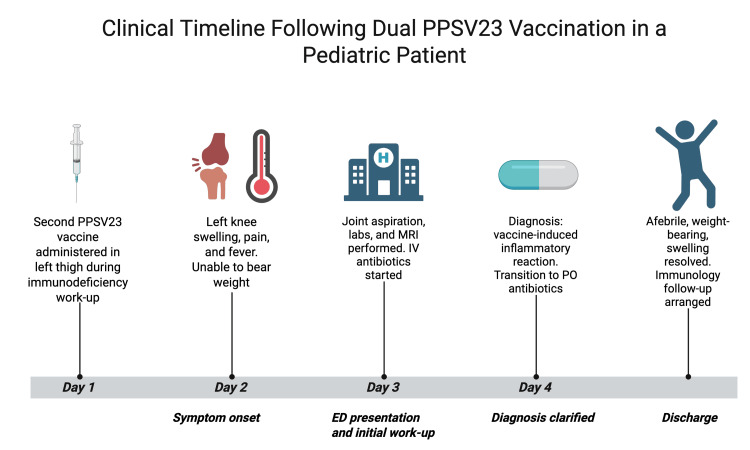
Cinical timeline summarizing key events including pneumococcal vaccination, symptom onset, diagnostic evaluation, and resolution. PPSV23: 23-valent pneumococcal polysaccharide vaccine; PO: per os (by mouth)

Upon discharge, the patient was afebrile, weight-bearing, and had a significant reduction in joint swelling. A follow-up visit with the immunology team was arranged to continue her evaluation for underlying immunodeficiency and to monitor for any complications. At the time of discharge, immunologic testing had been ordered by the immunology team, but results were not yet available. A visual timeline of clinical events is shown in Figure 2. 

## Discussion

The most frequently reported side effects of the PPSV23 in pediatric patients include fever, injection site erythema, and injection site pain, occurring in 43%, 28%, and 25% of cases, respectively [6]. Although these symptoms are expected post-vaccination, the team was initially unaware of her recent immunizations. Her inability to bear weight prompted further evaluation to rule out more serious pathology. Given the elevated leukocyte count within the patient’s synovial fluid with a neutrophilic predominance, the differential diagnosis included septic arthritis and transient synovitis. Transient synovitis was considered unlikely due to a lack of recent viral illnesses and milder expected inflammatory markers.

While the patient exhibited some features concerning for septic arthritis, including fever and elevated CRP, key findings were inconsistent with infection. The synovial WBC count was elevated but below the 50,000 cells/mm³ threshold commonly used to support a diagnosis of septic arthritis [7]. The high RBC count may have reflected a traumatic tap or mild hemarthrosis. The CBC differential showed neutrophilic predominance without bandemia or left shift. Additionally, Gram stain and cultures of synovial fluid and blood were both negative, favoring an inflammatory rather than infectious process. These findings, taken together, made septic arthritis less likely and guided the decision to manage the patient non-operatively. Although clinical decision tools such as the Kocher criteria are frequently referenced in suspected cases of septic arthritis, their utility is limited outside of the hip. In fact, one study found they missed up to 52% of septic knee cases, reinforcing the importance of joint-specific evaluation and clinical context when interpreting laboratory and imaging findings [8]. 

Vaccine-induced inflammatory reactions, though rare, can mimic septic arthritis and delay diagnosis. A review of vaccine-associated inflammatory myopathies notes that vaccines and viral infections may trigger immune responses, though causality remains unclear [9]. Emerging reports of myositis following COVID-19 vaccination, predominantly in older adults, mirror aspects of our patient’s presentation, including localized erythema, edema, and weakness after a second dose [10]. However, such reactions remain poorly defined in pediatric populations. Our case highlights the importance of including post-vaccination inflammatory reactions in the differential diagnosis for acute joint swelling in children, particularly in those with suspected or confirmed immunodeficiency, and calls attention to the need for further study in this area.

## Conclusions

The importance of obtaining a thorough vaccination history early in the evaluation of pediatric joint symptoms was emphasized by this case. This case also underscores the importance of maintaining a broad differential when evaluating acute joint symptoms in pediatric patients, particularly those undergoing immunodeficiency work-up. While septic arthritis remains a critical consideration, clinicians should remain vigilant for vaccine-induced inflammatory reactions, especially when there is a recent history of immunization. The clinical overlap between infectious and inflammatory processes can be striking, making timely recognition essential to avoid unnecessary interventions and to guide appropriate management.

As vaccine schedules become increasingly complex, this case highlights the need for careful coordination during immunologic assessments. Pediatric patients with underlying immune concerns may exhibit heightened responses to antigens, and closely spaced vaccinations may further amplify this effect. Improved awareness of these potential outcomes can inform clinical decision-making, reduce diagnostic uncertainty, and support continued confidence in vaccine safety.
